# Transition from Methylphenidate to Atomoxetine: reasons for switching and clinical outcome

**DOI:** 10.1192/bjo.2021.226

**Published:** 2021-06-18

**Authors:** Fabrizia Cassar, Giovanni Grech, Bertha Grech, Joseph Cassar

**Affiliations:** Mount Carmel Hospital

## Abstract

**Aims:**

Attention Deficit Hyperactivity Disorder (ADHD) is a behavior disorder originating in childhood comprising of a constellation of features including inattention, impulsivity, and hyperactivity. The National Institute of Clinical Excellence (NICE) Guidelines 2018 recommends methylphenidate as a first line pharmacological agent for treatment of children aged 5 years and over with ADHD. Lisdexamfetamine, dexamfetamine and atomoxetine are recommended in this order if methylphenidate is not tolerated or if symptoms did not respond to separate 6-week trials. Our aim was to, assess the transition of methylphenidate to atomoxetine, the reasons for switching and its clinical outcome in order to make recommendations to current practice regarding treatment of ADHD.

**Method:**

The study examined a total of 53 children between 0-16 years of age who were being treated for ADHD with atomoxetine at CYPS till September 2018. Data was collected from patients’ files retrospectively by using a proforma based on the NICE guidelines 2018 ADHD: diagnosis and management.

**Result:**

Out of 53 patients’ on atomoxetine in September 2018, 49 were included in the study. Results recorded side-effects as the main reason for switching from methylphenidate to atomoxetine. Unwanted side-effects were documented in 71.7% of patients of which 57.9% exhibited more than 1 side-effect with the two commonest side-effects documented being weight loss and decreased appetite. The audit highlighted the fact that the correct dose of atomoxetine was only administered in 17.2% of children with 56.9% of patient's being given a higher dose than recommended. Initial weight was not documented in 19% and hence, ideal dose could not be calculated. Overall, atomoxetine was shown to be an effective treatment. Out of the 40 patients documented to have hyperactivity this symptom was decreased in 82.5% whilst 82.9% were shown to have increased concentration. 35 patients had documented impulsivity and this was decreased in 62.9% of cases. 11 patients had documented anxiety with 72.7% being treated effectively with atomoxetine. 31% of patients’ had documented side-effects with 16% of these being tics. 20% of patient's required augmentation.

**Conclusion:**

The results indicate that the majority of doctors at CYPS in Malta adhered to the NICE guidelines 2018 and atomoxetine was proven to be efficacious as a second line drug in the treatment of ADHD. However, better adherence to NICE guidelines is required when it comes to the calculation of appropriate dosage. Our prediction is had dose recommendations according to weight been adhered to there may have been less side-effects documented.

